# Cerebral Neuroschistosomiasis Presenting as a Brain Mass

**DOI:** 10.7759/cureus.45418

**Published:** 2023-09-17

**Authors:** Salma Mustafa, Ahmad S Matarneh, Abdelaziz Mohamed, Abdalla Fadul, Muzamil Musa, Rowaa I Mohamed, Eltayeb Abdallah, Tameem G Mohamed

**Affiliations:** 1 Internal Medicine, Hamad Medical Corporation, Doha, QAT; 2 Radiology, Hamad Medical Corporation, Doha, QAT; 3 Internal Medicine, Hamad General Hospital, Doha, QAT

**Keywords:** headache, schistosoma, brain lesion, parasite infection, neuroschistosomiasis

## Abstract

Neuroschistosomiasis is a rare manifestation of schistosomal infections presenting with cerebral and spinal cord involvement. We reported a case of a 31-year-old woman who presented with a history of headache, dizziness, and nausea. Brain MRI with contrast showed features suggestive of brain lesion with edema, and a serology test for Schistosoma was positive. She was diagnosed with neuroschistosomiasis and treated with intravenous steroids followed by praziquantel resulting in a significant regression of the brain mass. Cerebral neuroschistosomiasis is a rare complication of Schistosoma infection, and clinicians should consider it among the differential diagnosis of unexplained brain lesions.

## Introduction

Schistosomiasis is an infectious helminthic disease commonly seen in the Middle Eastern region [1]. It is transmitted by contact with snails containing Schistosoma eggs and parasites. Snails, which are intermediate hosts, release cercariae into water. Cercariae, an infectious form of the Schistosoma parasite, penetrates the host skin, causing the infection [2]. Neuroschistosomiasis is a rare infection that was first discovered in 1930, with around 500 cases reported so far [3]. Neuroschistosomiasis usually involves the brain or the spinal cord, and the most commonly involved locations are the lumbosacral, cortex, subcortex, basal ganglia, and internal capsule [3]. We report a rare case of neuroschistosomiasis involving the cortical region manifested as a brain lesion with surrounding edema. Treatment with praziquantel and steroids has resulted in significant regression of the brain lesion.

## Case presentation

A 31-year-old female with a past history of hypertension presented to the hospital complaining of headache, dizziness, nausea, and vomiting for several weeks. She worked previously on a farm and was raising pigs while she was in the Philippines. She came to Qatar and currently works as a housekeeper. The physical examination showed a temperature of 36.5°C, blood pressure of 115/71 mmHg, and a heart rate of 87 beats/minute. On examination, she was fully alert and oriented. Neurological examination revealed normal muscle power, intact sensations, and preserved deep tendon reflexes. Fundoscopic examination of both eyes was also unremarkable. Basic laboratory investigations revealed high inflammatory markers. Renal function test indicated acute kidney injury, which improved to normal range after intravenous hydration. Basic laboratory investigations are summarized in Table 1.

**Table 1:** Basic laboratory investigations. INR: international normalized ratio

Variable	Value	Reference range
WBC	12×10^3^/μL	4.0-10.0×10^3^/μL
Hb	15.4 g/dL	13.0-17.0 g/dL
Platelet	400×10^3^/μL	150-400×10^3^/μL
Absolute neutrophil count	8×10^3^/μL	2.0-7.0×10^3^/μL
Neutrophil (%)	83.4%	-
INR	1.2	-
Urea	10.1 mmol/L	2.8-8.1 mmol/L
Creatinine	130 μmol/L	62-106 μmol/L
Bicarbonate	26 mmol/L	22-29 mmol/L
CRP	20 mg/L	0-5 mg/L

**Figure 1:**
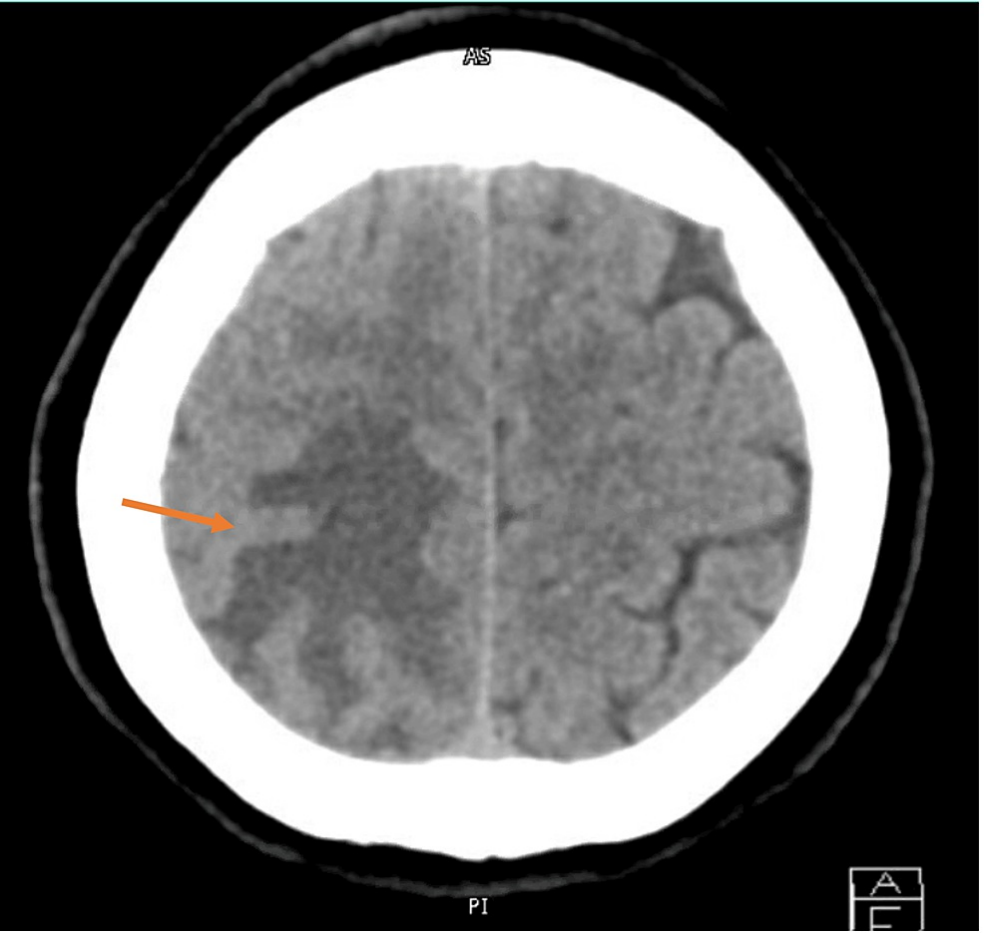
Right-sided occipitoparietal hypodensity with mass effect.

**Figure 2:**
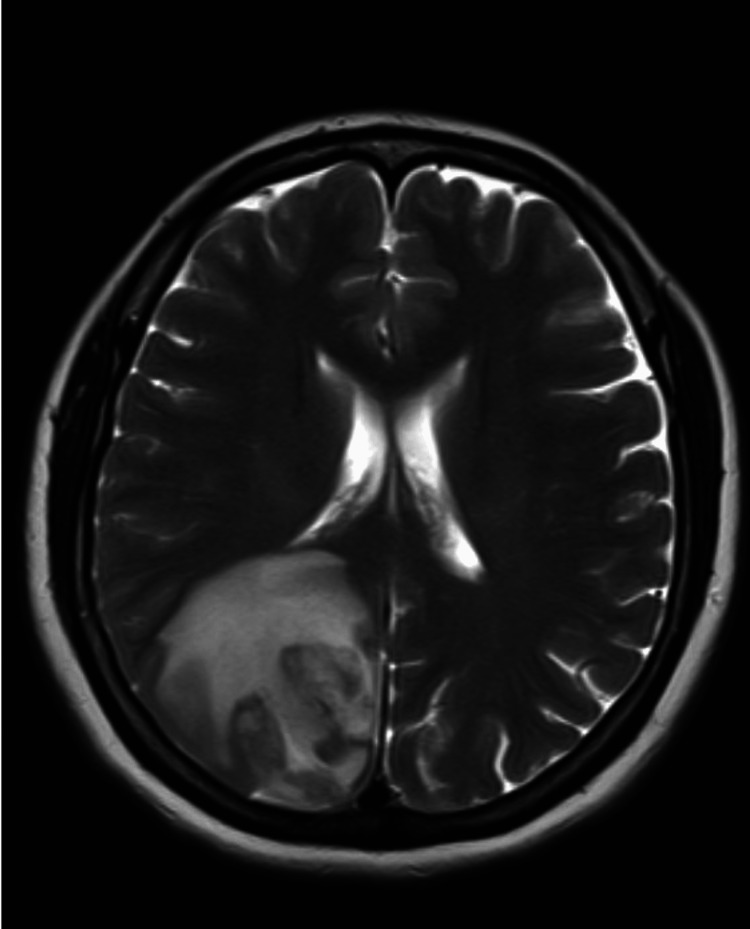
T2-weighted image showing right medial occipitoparietal cortical enhancing lesion with significant adjacent surrounding subcortical/white matter edema.

A plain computed tomography (CT) scan of the head showed ill-defined right occipitoparietal hypodensity with mass effect and suspicious right occipital density (Figure 1). She was started on intravenous dexamethasone to reduce the brain edema. Brain MRI revealed a right medial occipitoparietal essentially cortical irregular curvilinear/punctate enhancing lesion with significant adjacent surrounding subcortical/white matter edema (Figure 2). Lumbar puncture showed normal studies with a normal opening pressure of 18 cmH_2_O. Other investigations, including Toxoplasma IgG and IgM, CSF cryptococcal antigen, HIV serology, polymerase chain reaction for *Mycobacterium tuberculosis *(TB PCR), and stool for ova and parasite, were all negative.

**Figure 3:**
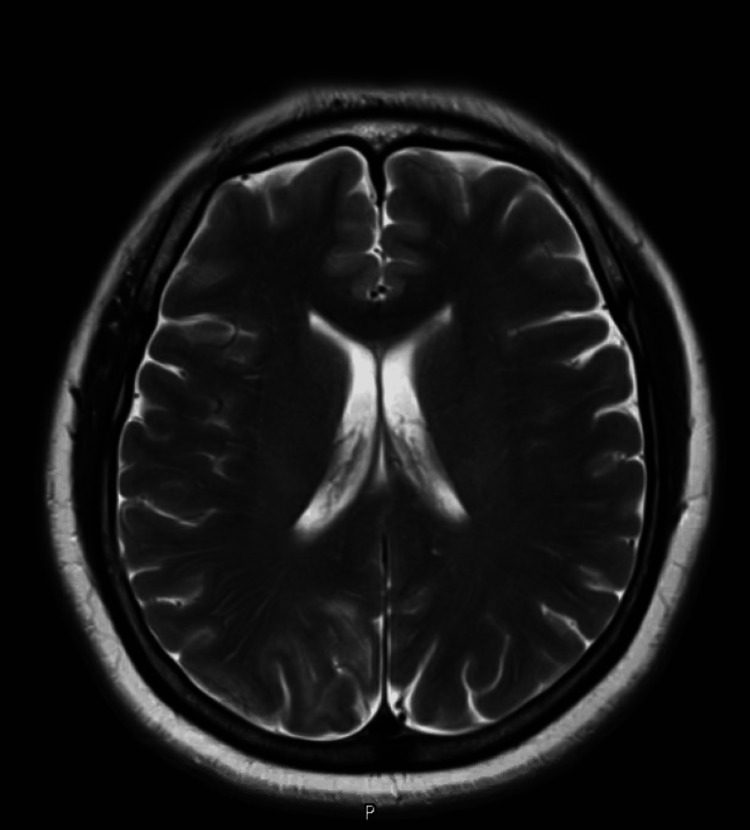
T2-weighted contrast-enhanced brain MRI showing significant regression of the previously noted right occipitoparietal lesion.

Schistosomiasis was suspected because of the typical MRI findings of punctate enhancement around the lesion with significant mass effect, her occupational history of working on a farm and exposure to pigs, and a negative workup for other infectious causes of brain lesions, e.g. toxoplasmosis and TB. Serology for Schistosoma antibodies came out positive with a titter of 1:160. Molecular PCR testing of schistosomiasis was not done because it is not available in our center. Brain biopsy was considered, but after discussion with the patient and involving a multidisciplinary team, the decision was made to treat her empirically and monitor the response without invasive brain biopsy mainly because of the high suspicion and the negative workup for other differential diagnoses. She was started on praziquantel 60 mg/kg every eight hours and received a total of four doses. Subsequently, the patient improved clinically and was discharged home on a tapering oral dexamethasone dose. Upon follow-up after two weeks, she stated complete resolution of the headache and dizziness. Repeated brain MRI after two weeks of treatment revealed significant regression of the previously noted brain lesion (Figure 3).

## Discussion

Schistosomiasis is a helminthic infection caused by Schistosoma flukes, and it is the second most common parasitic tropical disease after malaria [4]. Schistosomiasis is caused by Schistosoma species, such as *S. haematobium*, *S. japonicum*, *S. mansoni*, and *S. mekongi* [5]. Our patient was exposed in the Philippines before coming to Qatar. Schistosomiasis is endemic in 28 out of 81 provinces in the Philippines, with two million people directly at risk of schistosomiasis and 12 million living in endemic areas [6]. It can affect multiple organs, including the liver, intestines, urinary bladder, and central nervous system. Neuroschistosomiasis is a rare neurological infection with unknown pathophysiology [7]. The most accepted theory is that neuroschistosomiasis results from the embolization of the organism’s eggs to the central nervous system. Schistosoma eggs release proteolytic enzymes in the central nervous system, which induces a local eosinophilic inflammation. The resultant inflammation causes damage with granuloma formation and, eventually, fibrosis and demyelination of the surrounding structures. Neuroschistosomiasis is divided into cerebral schistosomiasis and spinal schistosomiasis [8]. Cerebral schistosomiasis is caused by *S. japonicum *and results in encephalitis, with headache, seizure, and altered mental status. On the other hand, spinal schistosomiasis is usually caused by *S. mansoni *(and less commonly *S. haematobium*) and causes myelitis with symptoms of weakness, back pain, and urine retention. 

Diagnosing neuroschistosomiasis can be challenging as it requires a high index of clinical suspicion. Brain MRI is the gold standard study to diagnose CNS involvement, and the characteristic findings of neuroschistosomiasis on MRI are single or multiple hyperintense lesions with punctate enhancement surrounded by edema with significant mass effect [9,10]. According to a 2013 study by Floriano et al., the differential diagnosis of infectious brain lesions that may mimic brain neoplasms includes tuberculosis, cysticercosis, pyogenic abscesses, toxoplasmosis, fungal infections, and syphilis. Authors stated that in patients with HIV, the most common brain mass lesion is toxoplasmosis, which is usually manifested as multiple, nodular, or ring-like enhancing lesions with surrounding vasogenic edema, located both in the white matter and deep gray matter [11]. Joy and Sakalecha in 2023 mentioned that the most common infectious causes of ring-enhancing brain lesions are neurocysticercosis and tuberculoma. They compared the MRI findings of 25 cases of neurocysticercosis and 17 cases of tubercloma and found that all cases of neurocysticercosis have thin ring enhancement compared to thick irregular ring enhancement in the majority of tuberclomas. The authors also mentioned that the use of multiparametric MRI assessment can help in prompt diagnosis and eliminate the need for brain biopsy [12].

A definitive diagnosis usually requires a brain biopsy, which is an invasive process. Serology is a sensitive test for diagnosing schistosomiasis but is limited by low specificity, resulting in a high false positivity rate. However, when the titers are high (1:160), they are considered significant [7,13]. Although brain biopsy is the gold standard for definitive diagnosis, we managed our patient empirically with praziquantel plus steroids based on her preference and the positive Schistosoma serology. The dramatic response seen on the follow-up MRI helped to confirm the diagnosis of neuroschistosomiasis.

After establishing the diagnosis, prompt treatment should be started to maximize the outcome. Steroids with a dose of 8 mg daily should be started before praziquantel to decrease the inflammation that might result from the cytotoxic effect of praziquantel and to be continued as tapering regimen [14]. Praziquantel with a dose of 60 mg/kg every 8 hours for three days duration should be given, it acts by causing tetanic contraction and paralyzing the parasite [15]. However, it acts only on the mature worms and not the larvae, making it ineffective in the early stages of infection [16]. Delayed treatment can result in complications which include intracranial hypertension, hydrocephalus, and myeloradiculopathy [17].

## Conclusions

Cerebral neuroschistosomiasis is one of the rare presentations of Schistosoma infection that can present with unexplained brain lesion and can result in severe irreversible complications. Early diagnosis and treatment are needed to prevent further deterioration. Treatment with steroids and praziquantel is usually the mainstay therapy.
